# Glycosylation of Vanillin and 8-Nordihydrocapsaicin by Cultured *Eucalyptus perriniana* Cells

**DOI:** 10.3390/molecules17055013

**Published:** 2012-05-02

**Authors:** Daisuke Sato, Yuki Eshita, Hisashi Katsuragi, Hiroki Hamada, Kei Shimoda, Naoji Kubota

**Affiliations:** 1 Department of Chemistry, Faculty of Medicine, Oita University, 1-1 Hasama-machi, Oita 879-5593, Japan; 2 Department of Infectious Disease Control, Faculty of Medicine, Oita University, 1-1 Hasama-machi, Oita 879-5593, Japan; 3 Sunny Health Co. Ltd., Yaesu k Bilg., 2-1-6 Yaesu, Chuo-ku, Tokyo 104-0028, Japan; 4 Department of Life Science, Okayama University of Science, 1-1 Ridai-cho, Okayama 700-0005, Japan

**Keywords:** biotransformation, glycosylation, vanilloid, plant cultured cells, *Eucalyptus perriniana*

## Abstract

Glycosylation of vanilloids such as vanillin and 8-nordihydrocapsaicin by cultured plant cells of *Eucalyptus perriniana* was studied. Vanillin was converted into vanillin 4-*O*-β-D-glucopyranoside, vanillyl alcohol, and 4-*O*-β-D-glucopyranosylvanillyl alcohol by *E. perriniana* cells. Incubation of cultured *E. perriniana* cells with 8-nor- dihydrocapsaicin gave 8-nordihydrocapsaicin 4-*O*-β-D-glucopyranoside and 8-nordihydro- capsaicin 4-*O*-β-D-gentiobioside.

## 1. Introduction

Biotransformations are considered to be an important method for converting inexpensive and plentiful organic compounds into costly and scarce ones [1,2,3,4,5]. In recent years, plant cell cultures have been studied as potential agents for biotransformation reactions according to the biochemical potential of plant enzymes to produce specific secondary metabolites [1]. Particularly, glycosylation by cultured plant cells has attracted much attention, because one-step enzymatic glycosylation by cultured plant cells is more convenient than chemical glycosylation which requires tedious protection-deprotection procedures [6,7,8,9,10,11,12,13].

Vanilloids including capsaicinoids have been widely used as food additives and have been studied for their broad range of physiological properties such as antioxidative, antimicrobial, analgesic, antigenotoxic, antimutagenic, and anticarcinogenic effects [14,15,16,17,18,19]. Irrespective of such biological and pharmacological activities, vanilloids are water-insoluble and poorly absorbable after oral administration. Furthermore, capsaicinoids are pungent principles of hot peppers, exhibiting direct skin and mucous membrane irritant effects [20]. These shortcomings have limited their use as food additives and medicines. Glycosylation allows water-insoluble and pungent organic compounds to be converted into the corresponding water-soluble and non-pungent compounds to improve their bioavailability. Here we report the glycosylation of vanillin and 8-nordihydrocapsaicin to their glucoconjugates, with greater water-solubility, by cultured plant cells of *Eucalyptus perriniana*.

## 2. Results and Discussion

### 2.1. Biotransformation of Vanillin

The biotransformation of vanilloids, *i.e.*, vanillin and 8-nordihydrocapsaicin, was individually investigated using cultured cells of *E. perriniana*. After addition of substrates to the cultured suspension cells in Murashige and Skoog’s (MS) medium, the incubation was continued for five days, after which products in each medium and MeOH extracts of cells were detected by HPLC. No products were obtained in the control experiments undertaken without substrate.

**Figure 1:**
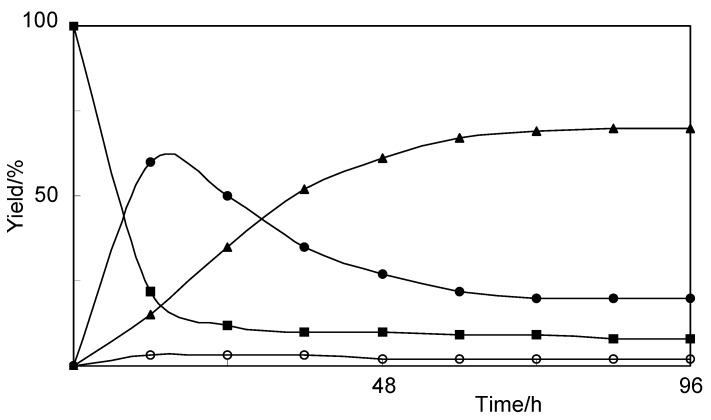
Time course of the biotransformation of vanillin (**1**) by cultured cells of *E. perriniana*. Yields of **1** (■), **2** (○), **3** (●), and **4** (▲) are plotted.

**Scheme 1:**
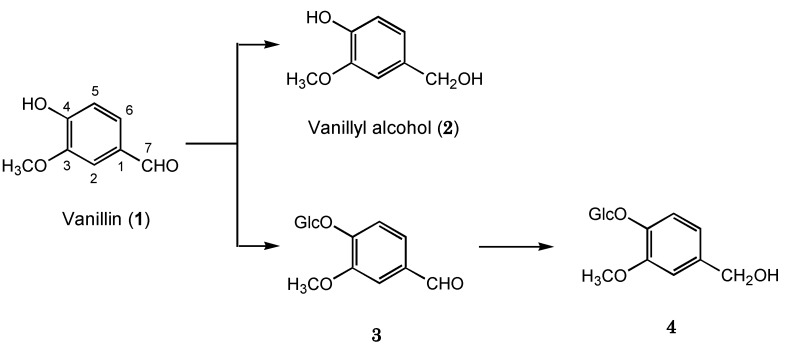
Biotransformation pathway of vanillin (**1**) by plant cultured cells of *E. perriniana*.

Incubation of vanillin (**1**) with the cultured *E. perriniana* cells for five days yielded compounds **2**–**4** which were identified as vanillyl alcohol (2%), vanillin 4-*O*-β-D-glucopyranoside (20%), and 4-*O*-β-D-glucopyranosylvanillyl alcohol (70%), respectively, by comparison of spectroscopic data, such as HRFABMS, ^1^H- and ^13^C-NMR (Table 1), H-H COSY, C-H COSY, and HMBC-spectra, with those reported previously [21,22]. A time course experiment (Figure 1) revealed that vanillin 4-*O*-β-D-glucopyranoside (**3**) was formed, followed by formation of 4-*O*-β-D-glucopyranosylvanillyl alcohol (**4**) (Scheme 1) because the amount of vanillin 4-*O*-β-D-glucopyranoside (**3**) decreased, accompanied by an increase of that of 4-*O*-β-D-glucopyranosylvanillyl alcohol (**4**). These findings suggest that vanillin 4-*O*-β-D-glucopyranoside (**3**) was converted into 4-*O*-β-D-glucopyranosylvanillyl alcohol (**4**) by *E. perriniana* cells.

**Table 1:** ^13^C chemical shifts of the glycosides **3**, **4**, **6**, and **7** in CD_3_OD.

Compound		3	4	6	7
Aglycone	1	130.2	136.0	134.8	135.1
	2	110.1	110.7	113.0	113.1
	3	151.4	148.4	150.5	150.8
	4	148.9	144.9	146.8	146.7
	5	114.2	114.8	117.9	118.3
	6	125.1	118.2	121.1	121.4
	7	191.2	62.5	43.6	43.8
	8			175.7	176.0
	9			37.0	37.1
	10			27.0	27.1
	11			30.3	30.3
	12			30.3	30.3
	13			30.3	30.3
	14			32.9	32.9
	15			23.6	23.7
	16			14.4	14.4
	OCH_3_	55.4	55.3	56.6	56.7
Glc	1'	99.1	99.9	102.7	102.5
	2'	72.8	73.0	74.7	75.1
	3'	76.9	76.7	77.9	77.8
	4'	69.3	69.4	71.2	71.5
	5'	76.6	76.6	77.6	77.6
	6'	60.3	60.4	62.4	69.4
	1''				104.3
	2''				74.8
	3''				77.6
	4''				71.3
	5''				77.8
	6''				62.6

### 2.2. Biotransformation of 8-Nordihydrocapsaicin

**Figure 2:**
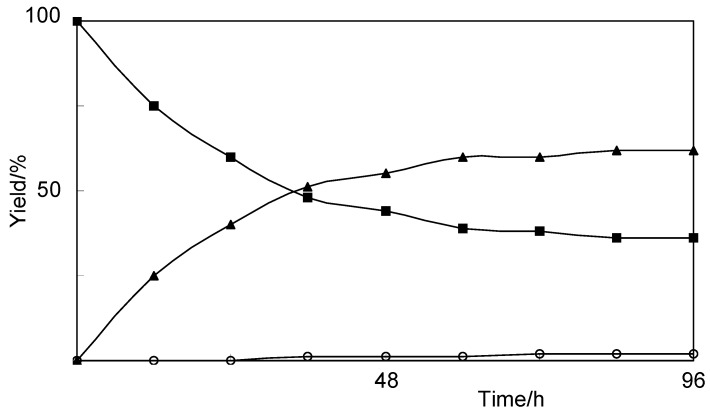
Time course of the biotransformation of 8-nordihydrocapsaicin (**5**) by cultured cells of *E. perriniana*. Yields of **5** (■), **6** (▲), and **7** (○) are plotted.

**Scheme 2:**
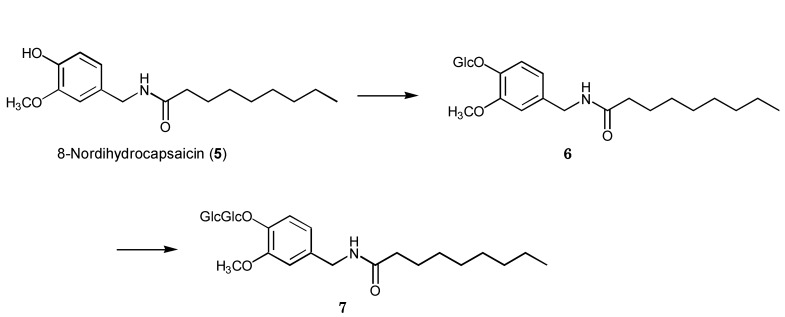
Biotransformation pathway of 8-nordihydrocapsaicin (**5**) by plant cultured cells of *E. perriniana*.

After cultured cells of *E. perriniana* were incubated with 8-nordihydrocapsaicin (**5**) for five days, compounds **6** and **7** were obtained as the products. They were identified as 8-nordihydrocapsaicin 4-*O*-β-D-glucopyranoside (62%) [23] and 8-nordihydrocapsaicin 4-*O*-β-D-gentiobioside (Glc-β-1,6-Glc) (1%), respectively. The time-course experiment results (Figure 2) indicated that 8-nordihydrocapsaicin 4-*O*-β-D-glucopyranoside was produced first and further glucosylation gave 8-nordihydrocapsaicin 4-*O*-β-D-gentiobioside, as shown in Scheme 2. The HRFABMS spectrum of cmpound **7** included a pseudomolecular ion [M+Na]^+^ peak at *m*/*z* 640.2920, consistent with a molecular formula of C_29_H_47_NO_13_ (calcd. 640.2903 for C_29_H_47_NO_13_Na). 

The ^1^H NMR spectrum of compound **7** included proton signals at *δ* 4.35 (1H, *d*, *J* = 7.2 Hz) and 4.92 (1H, *d*, *J* = 7.6 Hz), indicating the presence of two β-anomers in the sugar moiety. The ^1^H- and^ 13^C-NMR spectra of compound **7** indicated that it was a β-gentiobiosyl analogue of 8-nordihydrocapsaicin. Thus, the structure of compound **7** was determined as 8-nordihydrocapsaicin 4-*O*-β-D-gentiobioside, which has not been reported before.

## 3. Experimental

### 3.1. Analysis of the Products

The structures of the products were determined on the basis of analysis of their HRFABMS, ^1^H- and ^13^C-NMR, H-H COSY, C-H COSY, and HMBC spectra. The ^1^H- and ^13^C-NMR, H-H COSY, C-H COSY, and HMBC spectra were recorded using a Varian XL-400 spectrometer in CD_3_OD solution and the chemical shifts are expressed in *δ* (ppm) referring to TMS. The HRFABMS spectra were measured using a JEOL MStation JMS-700 spectrometer. 

### 3.2. Cell Line and Culture Conditions

Cultured *E. perriniana* cells were subcultured at 4-week intervals on solid Murashige and Skoog (MS) medium (100 mL in a 300-mL conical flask) containing 3% sucrose, 10 mM 2,4-dichlorophenoxyacetic acid, and 1% agar (adjusted to pH 5.7) at 25 °C in the dark [24]. A suspension culture was started by transferring the cultured cells to 100 mL of liquid medium in a 300-mL conical flask, and incubated on a rotary shaker (120 rpm) at 25 °C in the dark. Prior to use for this work, part of the callus tissues (fr. wt 40 g) was transplanted to freshly prepared MS medium (100 mL in a 300-mL conical flask) and grown with continuous shaking for 2 weeks on a rotary shaker (120 rpm).

### 3.3. Biotransformation and Purification of Products

To the 500 mL flask containing 200 ml of MS medium and the suspension cultured cells (100 g) of *E. perriniana* was added 15 mg of substrate. The cultures were incubated at 25 °C for 5 days on a rotary shaker (120 rpm) in the dark. After the incubation period, the cells and medium were separated by filtration with suction. Extraction and purification procedures of biotransformation products were performed according to the previously reported methods [9,10,11,12,13]. The yield of the products was determined on the basis of the peak area from HPLC [column: YMC-Pack R&D ODS column (150 × 30 mm); solvent: MeOH-H_2_O (9:11, v/v); detection: UV (280 nm); flow rate: 1.0 mL/min] and expressed as a relative percentage to the total amount of the whole reaction products extracted.

*8-Nordihydrocapsaicin 4-O-β-D-gentiobioside* (2 mg): HRFABMS: *m*/*z* 640.2920 [M+Na]^+^ (calcd 640.2903 for C_29_H_47_NO_13_Na); ^1^H-NMR (CD_3_OD): *δ* 0.89 (3H, t, *J* = 6.8 Hz, H-16), 1.31 (10H, m, H-11, 12, 13, 14, 15), 1.63 (2H, q, *J* = 7.6 Hz, H-10), 2.24 (2H, t, *J* = 7.6 Hz, H-9), 3.10–4.11 (12H, m, H-2', 2'', 3', 3'', 4', 4'', 5', 5'', 6', 6''), 3.85 (3H, s, OCH_3_), 4.31 (2H, d, *J* = 8.0 Hz, H-7), 4.35 (1H, d, *J* = 7.2 Hz, H-1''), 4.92 (1H, d, *J* = 7.6 Hz, H-1'), 6.85 (1H, dd, *J* = 8.0, 1.6 Hz, H-6), 6.94 (1H, s, H-2), 7.15 (1H, d, *J* = 8.4 Hz, H-5); ^13^C-NMR (CD_3_OD), see Table 1.

## 4. Conclusions

The results of this experiment revealed that the cultured suspension cells of *E*. *perriniana* are able to convert vanilloids, including capsaicinoid, into the corresponding β-glucosides and β-gentiobioside. Recently, several attempts have been made to glycosylate vanillin by cultured plant cells that resulted in the production of only vanillin 4-*O*-β-D-glucopyranoside [21,22]. We have already reported that cultured plant cells of *E. perriniana* had high potential to glycosylate exogenous compounds to their glycosides including gentiobiosides [10,12,13]. However, there have been no reports on the glycosylations of vanillin to the glycoside of vanillyl alcohol and of 8-nordihydrocapsaicin to its gentiobioside derivative, and the biotransformation pathway of vanilloids in cultured plant cells has not yet been elucidated. This paper reports, for the first time, the biological production by cultured plant cells of vanilloid glycosides, such as β-glucoside of vanillyl alcohol and 8-nordihydrocapsaicin β-gentiobioside, which was more soluble [25]. The biotransformation pathway of vanillin to β-glucoside of vanillyl alcohol has not been reported so far. Recently, a few studies were reported on the biocatalytic synthesis of capsaicin primeveroside and vicianoside using *Catharanthus roseus* cells [26], and on chemo-enzymatic synthesis of dihydronorcapsaicin β-D-glucopyranoside [27]. Cultured *C. roseus* cells are more active rather than *E. perriniana* cells in the capsaicin glycosylations. Further studies on the pharmacological activities of vanilloid glycosides are now in progress.

## Supplementary Materials

Supplementary materials can be accessed at http://www.mdpi.com/1420-3049/17/5/5013/s1.
