# Supplementary figures and images for: Glycosylation of Vanillin and 8-Nordihydrocapsaicin by Cultured Eucalyptus perriniana Cells
Source: Molecules. 2012 May 2;17(5):5013–20. doi: 10.3390/molecules17055013 (PMC6268922; doi:10.3390/molecules17055013)

# Supplementary Materials

## $^1\text{H}$ -NMR spectra of Compound 7

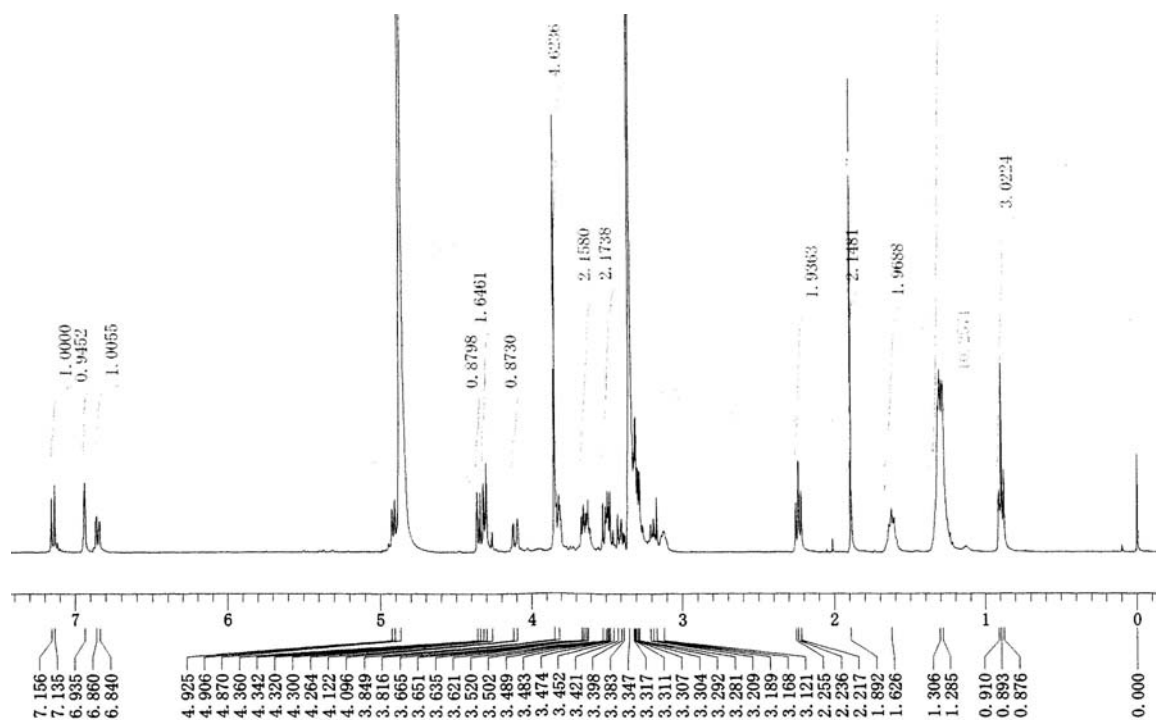

## $^{13}\text{C}$ -NMR spectra of Compound 7

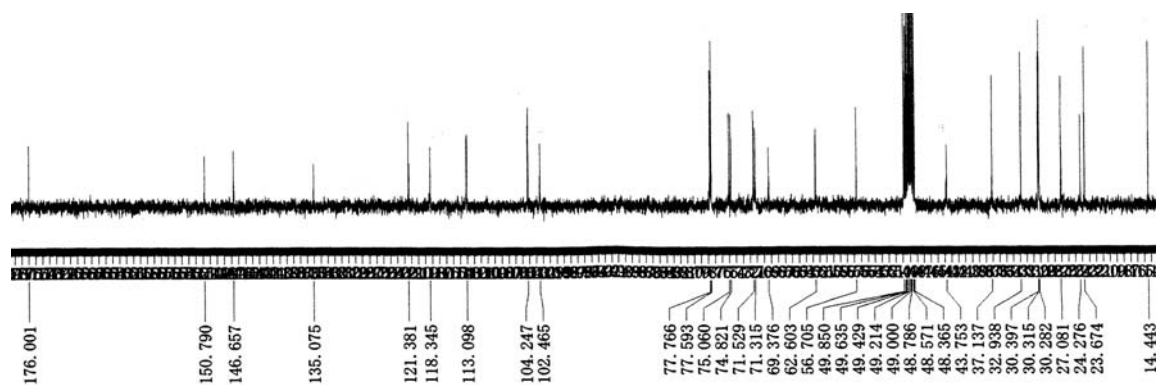

Supplement: Supplementary file 1 [file molecules-17-05013-s001.pdf]
